# Grapevine acclimation to water deficit: the adjustment of stomatal and hydraulic conductance differs from petiole embolism vulnerability

**DOI:** 10.1007/s00425-017-2662-3

**Published:** 2017-02-18

**Authors:** Uri Hochberg, Andrea Giulia Bonel, Rakefet David-Schwartz, Asfaw Degu, Aaron Fait, Hervé Cochard, Enrico Peterlunger, Jose Carlos Herrera

**Affiliations:** 1 Department of Agricultural, Food, Environmental and Animal Sciences, University of Udine, Via delle Scienze 206, 33100 Udine, Italy; 2 PIAF, INRA, Univ. Clermont-Auvergne, 63100 Clermont-Ferrand, France; 3 Institute of Plant Sciences, Agricultural Research Organization, The Volcani Centre, 50250 Bet Dagan, Israel; 4 The French Associates Institute for Agriculture and Biotechnology of Drylands, Ben Gurion University of the Negev, Sede Boqer, Israel; 5 Division of Viticulture and Pomology, Department of Crop Sciences, University of Natural Resources and Life Sciences Vienna (BOKU), Konrad Lorenz Str. 24, 3430 Tulln, Austria

**Keywords:** Drought acclimation, Osmotic adjustment, Turgor, Vulnerability to cavitation, Water stress, Xylem architecture

## Abstract

**Drought-acclimated vines maintained higher gas exchange compared to irrigated controls under water deficit; this effect is associated with modified leaf turgor but not with improved petiole vulnerability to cavitation.**

A key feature for the prosperity of plants under changing environments is the plasticity of their hydraulic system. In the present research we studied the hydraulic regulation in grapevines (*Vitis vinifera* L.) that were first acclimated for 39 days to well-watered (WW), sustained water deficit (SD), or transient—cycles of dehydration–rehydration—water deficit (TD) conditions, and then subjected to varying degrees of drought. Vine development under SD led to the smallest leaves and petioles, but the TD vines had the smallest mean xylem vessel and calculated specific conductivity (*k*_ts_). Unexpectedly, both the water deficit acclimation treatments resulted in vines more vulnerable to cavitation in comparison to WW, possibly as a result of developmental differences or cavitation fatigue. When exposed to drought, the SD vines maintained the highest stomatal (*g*_s_) and leaf conductance (*k*_leaf_) under low stem water potential (Ψ_s_), despite their high xylem vulnerability and in agreement with their lower turgor loss point (Ψ_TLP_). These findings suggest that the down-regulation of *k*_leaf_ and *g*_s_ is not associated with embolism, and the ability of drought-acclimated vines to maintain hydraulic conductance and gas exchange under stressed conditions is more likely associated with the leaf turgor and membrane permeability.

**Electronic supplementary material:**

The online version of this article (doi:10.1007/s00425-017-2662-3) contains supplementary material, which is available to authorized users.

## Introduction

The morphology, anatomy, and physiology of the entire hydraulic path of the plant, from the root hairs up to the stomata, will define the plant’s capacity to uptake, transport and transpire water (Sperry et al. 2002). A change in water availability or demand will be followed by a quick (minutes) regulation of the transpiration. If the new environmental condition is maintained for a longer period (days), the plant will slowly modify its hydraulic system to improve its compatibility with the changing environment (i.e. hydraulic acclimation; Lovisolo et al. 2010).

The fastest regulation of transpiration occurs at the leaf level, where the gradient of water potentials is the largest (Van den Honert 1948). The importance of stomatal regulation of transpiration has been acknowledged for over a century (Darwin 1898), but evidence from the last decade suggests that leaf hydraulic conductance (*k*_leaf_) is also an important regulator of transpiration, possibly through amplification of the hydraulic signal (Sack and Holbrook 2006; Brodribb et al. 2007; Scoffoni et al. 2014). In response to declining soil water content (*θ*) and stem water potential (Ψ_s_), both stomatal conductance (*g*_s_) and *k*_leaf_ are quickly down-regulated, resulting in immediate decline of transpiration.

Conversely, plant acclimation to water deficit involves slower processes, including the modification of fundamental hydraulic characteristics. To begin with, the most noticeable effect is the modification of leaf area (i.e. the evaporative surface), either through growth seizure, or even by the shedding of leaves. Additionally, evidence for a broad hydraulic adjustment showed that during drought acclimation the size of xylem vessels and their conductive capacity is normally smaller (Lovisolo and Schubert 1998), xylem resistance to cavitation is improved (Awad et al. 2010; Fichot et al. 2010), and osmolites accumulation is increased leading to lower turgor loss point (Ψ_TLP_; Patakas et al. 1997). These acclimations to drought mirror the characteristics that are common to species adapted to arid environments (Kolb and Sperry 1999; Brodribb et al. 2003; Bartlett et al. 2014), and enable the plant to sustain its new surroundings.

Assuming that such hydraulic adjustments occur during acclimation to water deficit, the mechanical interaction between *θ*, Ψ_s_, turgor pressure, *k*_leaf_, and *g*_s_ should be modified following the acclimation. Namely, the fast control of transpiration should be modified in respect to the slow changes the hydraulic system underwent during the acclimation period. For example, acclimation leading to smaller vessels should subsequently result in higher resistance of the xylary pathway, leading to either a larger gradient between the soil and leaf water potential (Ψ_l_), or reduction of the transpiration rates. Similarly, acclimation leading to lower Ψ_TLP_ should allow vines to maintain higher *g*_s_ under similar Ψ_s_ (Scarth 1927). These modifications are expected to be finely tuned to assure that the adjustment of stomatal regulation leads to tensions that the xylem could sustain without danger of embolism propagation. Nonetheless, data on the interaction between the fast stomatal regulation and the slow hydraulic modification during acclimation to drought is lacking. The present experiment was designed to evaluate the effect of acclimation on drought stress responses by first subjecting grapevines to a drought acclimation period and then exposing the drought-acclimated vines to a successive water deficit. We hypothesized that the adjustments developed during the acclimation (e.g. modified xylem architecture, xylem vulnerability to cavitation, and Ψ_TLP_) will cause changes in *k*_leaf_ and *g*_s_ regulation in a manner that both allows gas exchange and retains the hydraulic integrity of the xylem under future low Ψ conditions.

## Materials and methods

**Fig. 1:**
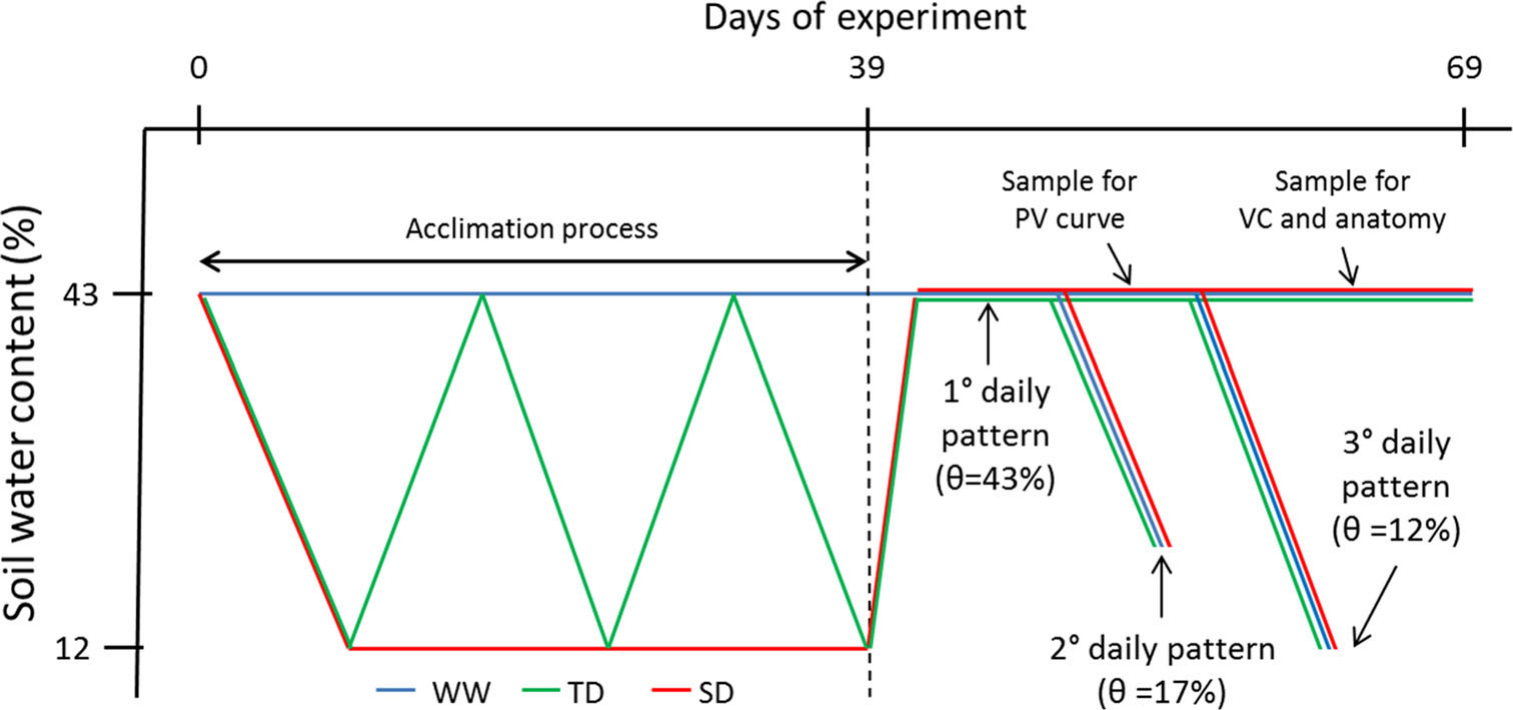
Description of the experimental timeline for the well-watered (WW), transient deficit (TD), and sustained deficit (SD) treatments. Following the 39-day acclimation period, the daily pattern of stomatal conductance and water potential under three different volumetric soil water contents (*θ*) were evaluated on three different days. Additionally, pressure volume curve (PV), the petiole xylem vulnerability curve (VC), and the petiole anatomy were evaluated in the plants acclimated to different treatments

This study consisted of two main experiments: (1) a 39-day acclimation period to drought, and (2) a dehydration experiment on the drought-acclimated vines (schematized in Fig. 1). During the 39-day acclimation period, grapevines were subjected to three different irrigation treatments: sustained water deficit (SD), transient water deficit—three cycles of dehydration–rehydration—(TD), and well-watered (WW). Treatments during the acclimation period were designed to simulate what might happen in open fields, where growing plants could experience abundant water availability, little water availability, or cycles of stress and recovery (for instance when irrigation is performed once a week). The soil volumetric water content (*θ*), leaf abscisic acid concentration (ABA), and stomatal conductance (*g*_s_) were used to characterize the degree of water stress for each of the acclimation treatments. Following the 39-day acclimation period, all plants were rehydrated and maintained under well-watered conditions for a month in order to avoid long-term influence of chemical signaling (i.e. ABA). ABA is accumulated in leaves during drought and is responsible for maintaining stomata closed even after re-watering until it is completely metabolized (Tombesi et al. 2015). During this period, the adjustment of leaf physiology was evaluated for each treatment through various measurements: leaf pressure volume curves, petiole vulnerability curves, and cross-section anatomy. Finally, in order to observe the leaves’ hydraulic regulation resulting from acclimation, plants were subjected to a second water deficit experiment. Leaf water potential (Ψ_l_), stem water potential (Ψ_s_), and stomatal conductance (*g*_s_) were measured on three different days. For each of these 3 days, the soil was maintained at different *θ*, reflecting different degrees of water stress.

### Growth conditions and irrigation treatments

The experiment took place in the greenhouse complex at the University of Udine (northeastern Italy). The experiment was conducted on 150 cuttings of *Vitis vinifera* cv. Merlot (clone R3) grafted on SO_4_ (*V. berlandieri* × *V. riparia* cv. ISV-VCR4) rootstock (Vivai Cooperativi Rauscedo VCR, Rauscedo, Italy) and planted in January 2015 in 7 L pots filled with exactly 1.2 kg (dry weight) of commercial potting media (Gebr. Brill Substrate Type 1, Georgsdorf, Germany). Budbreak occurred on 1 March 2015 and only one shoot per plant was left to develop vertically. The temperature and humidity were continuously monitored (Vaisala HMP45C, Helsinki, Finland) with average daily temperature ranging from 12.8 to 26.1 °C (Fig. S1).

The vines were irrigated for a period of 39 days according to the three different treatments. Each treatment had 50 vines. Pots were arranged in a completely randomized design. Irrigation treatments started when the vines had three mature leaves, 1 month after budbreak, and until then all pots were irrigated as the well-watered treatment. The WW treatment was drip-irrigated three times a day, with a gradual increase of irrigation volumes (0.5–1.5 L day^−1^ according to plant growth and water demand) to maintain *θ* near field capacity. This high frequency of irrigation ensured that the pot water capacity was not a limiting factor. In the SD treatment, irrigation was withheld to deplete the soil water content until vines reached mid-morning *g*_s_ values of 0.05–0.15 mol H_2_O m^−2^ s^−1^. These mid-morning *g*_s_ values both reflect moderate stress conditions and allows the vines to maintain growth (Flexas et al. 2002). Once the desired *g*_s_ was reached—on the 12th day after the initiation of the irrigation experiment (DOE)—pots were manually irrigated on a daily basis using a measuring beaker to maintain a constant pot weight (scale max 120 kg, reading unit: 10 g) that should lead to a similar rate of *g*_s_. Irrigation values were between 50–100 mL day^−1^ for the SD treatment. The transient water deficit treatment (TD) underwent three dehydration–rehydration cycles. In each cycle, irrigation was withheld to deplete *θ* until *g*_s_ levels of 0.05–0.15 mol H_2_O m^−2^ s^−1^ were measured (i.e. as in SD), and irrigated immediately after, similar to the WW treatment for 6 days. Following the 39 days of different treatments, all plants were irrigated as the WW treatment until their physiological adjustments were evaluated as described below.

To assess the response of the vines to drought following the acclimation period, the vines’ daily course of *g*_s_ and Ψ_s_ were measured on three different days. On each of these days, the pots were dried to different *θ* (i.e. 43, 17, and 12%), which corresponded to no stress, moderate stress, and severe stress conditions, respectively. Specifically, daily patterns were measured 6 (no stress), 16 (moderate stress), and 19 (severe stress) days after the end of the acclimation period (Fig. 1). Different vines were measured on each day and at each time point (three plants per treatment at each time point). Therefore, the measured vines were not exposed to stress between the end of the acclimation period and the day on which they were measured. Moreover, because the vines from different treatments had different leaf areas (resulting in different transpiration rates) that might lead to differences in *θ* of their respective pots, each pot was manually irrigated every 2 h to maintain a constant weight and assure that similar *θ*’s were experienced by all the vines during the whole day of measurements.

### Leaf area and vine mass

To assess the vine growth during the acclimation period, total plant leaf area was measured on the 1st and 41st DOE. The leaf area of three vines per treatment was destructively measured at each time point, using a leaf area meter (Li-Cor LI-3100). Additionally, the above-ground current year growth of the same vines was weighed to determine the fresh weight and dry weight (after 5 days at 70 °C).

### Gas exchange, water potential (Ψ) and hydraulic conductance (*k*)

Measurements of gas exchange and water potential (Ψ, MPa) were performed on fully expanded leaves. To measure the water potential, the leaves were bagged immediately before (for leaf water potential, Ψ_l_) or 1 h before (for stem water potential, Ψ_s_) they were excised from the shoot using a sharp blade. While still bagged, the leaves were placed into the pressure chamber (Soil Moisture Co., Santa Barbara, CA, USA) with the petiole protruding from the chamber lid. The chamber was pressurized using a nitrogen tank, and Ψ was recorded when the initial xylem sap was observed emerging from the cut end of the petiole. Pre-dawn water potential (Ψ_PD_) was measured as Ψ_s_ 30 min before first light.

The LI-6400 portable photosynthesis system (Li-Cor Inc, NE, US) device was used to measure *g*_s_ (mol H_2_O m^−2^ s^−1^) and *E* (mmol H_2_O m^−2^ s^−1^) using ambient humidity and temperature.

The leaf hydraulic conductance (*k*_leaf_ specific to leaf area; Eq. 1) and whole plant hydraulic conductance (*k*_plant_ specific to leaf area; Eq. 2) were calculated based on the Ohm’s law analogy as following:

1 $$ k_{\text{leaf}} = \frac{E}{{\varPsi_{\text{s}} - \varPsi_{\text{l}} }}, $$

2 $$ k_{\text{plant}} = \frac{E}{{\varPsi_{\text{PD}} - \varPsi_{\text{l}} }}. $$

### Xylem vulnerability curves and pressure volume curves

To assess the physiological adjustments the vines underwent during the acclimation period, pressure volume curves (PV) and xylem vulnerability curves (VC) were performed 10–15 days (for PV) or 20–30 days (for VC) after the end of the acclimation period on petioles or leaves, respectively, that were differentiated during the acclimation period (i.e. after imposing different irrigation treatments). Different growth rates could alter the relationship between leaf age and development (Rapaport et al. 2014). Hence, since much faster growth was noted in the WW treatment (Table 1), it probably had a larger proportion of younger leaves (in days) and possibly a lower proportion of developed leaves. The potential effect of such differences, which are common in most long-term stress experiments, was controlled by selecting leaves of similar age. To know the differentiation time of the leaves, the highest internode bearing a 3 cm length leaf was marked on the 12th day of the experiment, when both the SD and TD treatment reached the desired low *g*_s_ values. The three leaves above the mark were used for these analyses.

**Table 1:** Total leaf area per vine (LA), plant dry weight, single leaf area (LA), and number of leaves per vine (#leaves) of the well-watered (WW), transient water deficit (TD), and sustained water deficit (SD) acclimation treatments on the 1st and 41st day of experiment (DOE)

DOE	Treatment	Total LA (cm^2^ vine^−1^)	Dry weight^*^ (g vine^−1^)	Single LA (cm^2^ leaf^−1^)	No. of leaves per vine
1	WW	268.4 ± 37.2^a^	1.2 ± 0.2^a^	57.2 ± 2.2^a^	7.3 ± 1.45^a^
	TD	327.0 ± 17.3^a^	1.4 ± 0.1^a^	64.6 ± 3.4^a^	7.0 ± 0.10^a^
	WD	277.4 ± 23.7^a^	1.4 ± 0.02^a^	56.1 ± 3.8^a^	6.7 ± 0.33^a^
41	WW	3147.3 ± 394.2^a^	27.9 ± 2.3^a^	119.0 ± 5.8^a^	16.7 ± 1.45^a^
	TD	2570.0 ± 205.5^b^	19.4 ± 1.8^b^	109.2 ± 1.1^b^	15.0 ± 1.15^b^
	WD	1205.6 ± 49.9^c^	8.5 ± 0.9^c^	94.9 ± 3.3^c^	9.3 ± 0.67^c^

Values are mean ± SE (*n* = 3). Different letters within a column and for each DOE indicate significant differences between treatments (*P* < 0.05) by Tukey Honest Significant Difference (HSD) test

* Only the current year growth was measured.

Xylem vulnerability curves were acquired exactly as described in the protocol presented by Hochberg et al. (2016b). Briefly, the vines were cut at their rootstock and dehydrated to a range of stem water potentials. Following dehydration the vines were covered with large black plastic bags for at least 20 min to allow water pressure stabilization. Two leaves per shoot were measured and averaged to determine Ψ_s_ and the shoot was submerged under 4 cm of water for 20 min to relax the tension. Hochberg et al. (2016b) showed that short xylem relaxation does not lead to refilling and allows an accurate evaluation of embolism. Following xylem relaxation, three petioles (4 cm long) per shoot were cut under water and connected to the Xyle’m hydraulic apparatus (Bronkhorst, Montigny-lès-Cormeilles, France). Distilled and degassed water, supplemented with 10 mM KCl, was used as perfusion liquid for all measurements. The initial hydraulic conductance (*k*_i_) was determined with a hydrostatic pressure gradient of 4–5 kPa. To measure the maximum conductance (*k*_max_), the petioles were flushed for a period of 2 min with water pressurized at 0.18 MPa. PLC was calculated as:

3 $$ {\text{PLC}} = 100 \times \left( {1 - \frac{{k_{i} }}{{k_{\hbox{max} } }}} \right). $$

The pressure volume (PV) curves were acquired as described in Turner (1988). Leaf weight and, immediately after, leaf water potential (Ψ_l_, MPa), were periodically measured during leaf dehydration. The turgor loss point (Ψ_TLP_), osmotic potential at 100% turgor (*π*_100_), and cell modulus of elasticity (*ε*) were estimated through the analysis of the PV curve (Turner 1988).

### Xylem architecture and theoretical specific hydraulic conductivity

The xylem architecture of seven of the petioles used for the xylem vulnerability curve analysis was examined. Samples were incubated in 70% ethanol and stored at 4 °C until dehydration. Fixed tissue was dehydrated at room temperature in a graded series of ethanol (1 h each at 70, 90, 95 and 100%). After dehydration, the samples were placed in small plastic net boxes and incubated in isopropyl alcohol inside a microwave histoprocessor (Milestone Histo5, Kalamazoo, MI, USA) for 105 min at 70 °C. To ensure adequate removal of isopropyl alcohol, a vacuum drying step was introduced, where the pressure was 660 mbar. Wax impregnation was performed under vacuum condition (10 min 70 °C 500 mbar, 10 min 70 °C 400 mbar, 4 min 70 °C 300 mbar, 4 min 70 °C 200 mbar, 3 min 70 °C 150 mbar, 89 min 65 °C 100 mbar). Cross-sections of 15 µm in thickness were prepared using a rotary microtome (Leica, RM2245), floated in water at 42 °C to stretch ribbons and incubated on microscope slides at 42 °C overnight. The sections were then stained with Safranin-O and Fast-green. The sectioned material was observed using a Leica IM1000 microscope, and digital images were taken using a CCD camera DC2000 (Leica, Wetzlar, Germany).

Since petioles are nearly symmetrical, half of the vessels (later extrapolated to the whole petiole) were manually (i.e. without an automated algorithm) labeled, and their areas were measured using the appropriate function of the ImageJ software (Abràmoff et al. 2004). Based on the radius of each vessel, theoretical hydraulic conductivity (*k*_t_; mmol m s^−1^ MPa^−1^) was calculated with the modified Hagen–Poisseuille’s law described by Tyree and Ewers (1991):

4 $$ k_{\text{t}} = \frac{\pi \rho }{128\eta }\mathop \sum \limits_{i = 1}^{n} (d_{i}^{4} ), $$

where *d* is the diameter of the vessel in meters, *ρ* is the fluid density (assumed to be 1000 kg m^−3^) and *η* is the viscosity (assumed to be 1 × 10^−9^ MPa s). The theoretical specific hydraulic conductivity (*k*_ts_) was calculated by normalizing *k*_t_ to the leaf area (LA): *k*_ts_ = *k*_t_/LA. The relative proportion of the number of vessels belonging to different size categories and their contribution to *k*_t_ was evaluated.

### Abscisic acid

Abscisic acid (ABA), a known marker for water stress, was monitored in all treatment during the acclimation period to assure that the 6 days of hydration allowed the TD to eliminate signs of stress. One leaf per plant for four plants per treatment (*n* = 4) was detached and immediately frozen under liquid nitrogen on the 13th, 18th, 29th, 34th, and 39th DOE, corresponding to the maximum and minimum peaks of the dehydration–rehydration cycles of TD. The leaves were maintained under −80 °C for 60 days before they were extracted and analyzed using UPLC-QTOF-MS system (Waters Q-TOF XevoTM, Waters MS Technologies) exactly as described in Hochberg et al. (2013). ABA concentration was determined using a standard calibration curve of ±ABA (OlChemIm, Olomouc, Czech Republic).

### Statistical analysis

In order to evaluate the Ψ_s_ in which 50% PLC is measured (PLC_50_), vulnerability curve data were fit to an exponential sigmoidal regression as follows:

5 $$ {\text{PLC}} = \frac{100}{{1 + e^{{\alpha (\varPsi_{\text{s}} - {\text{PLC}}_{50} )}} }}. $$

To test for significant differences between treatments, coefficients *α* and PLC_50_ were determined from the linearized form of Eq. 5, as follows:

6 $$ \ln (100/{\text{PLC}} - 1) = \alpha \times (\varPsi_{\text{s}} - {\text{PLC}}_{50} ). $$

Transformed values were analyzed through a regression model with Ψ_s_, the treatment, and their interaction as model effects.

Other parameters of the different treatment were averaged and compared using a Tukey HSD test (*P* < 0.05; JMP 7.0, SAS Institute, Cary, NC, USA). Differences and relationships were considered significant at *P* < 0.05. Figures were created using Sigmaplot 12.5 (Systat Software Inc.).

## Results

### Acclimation period

**Fig. 2:**
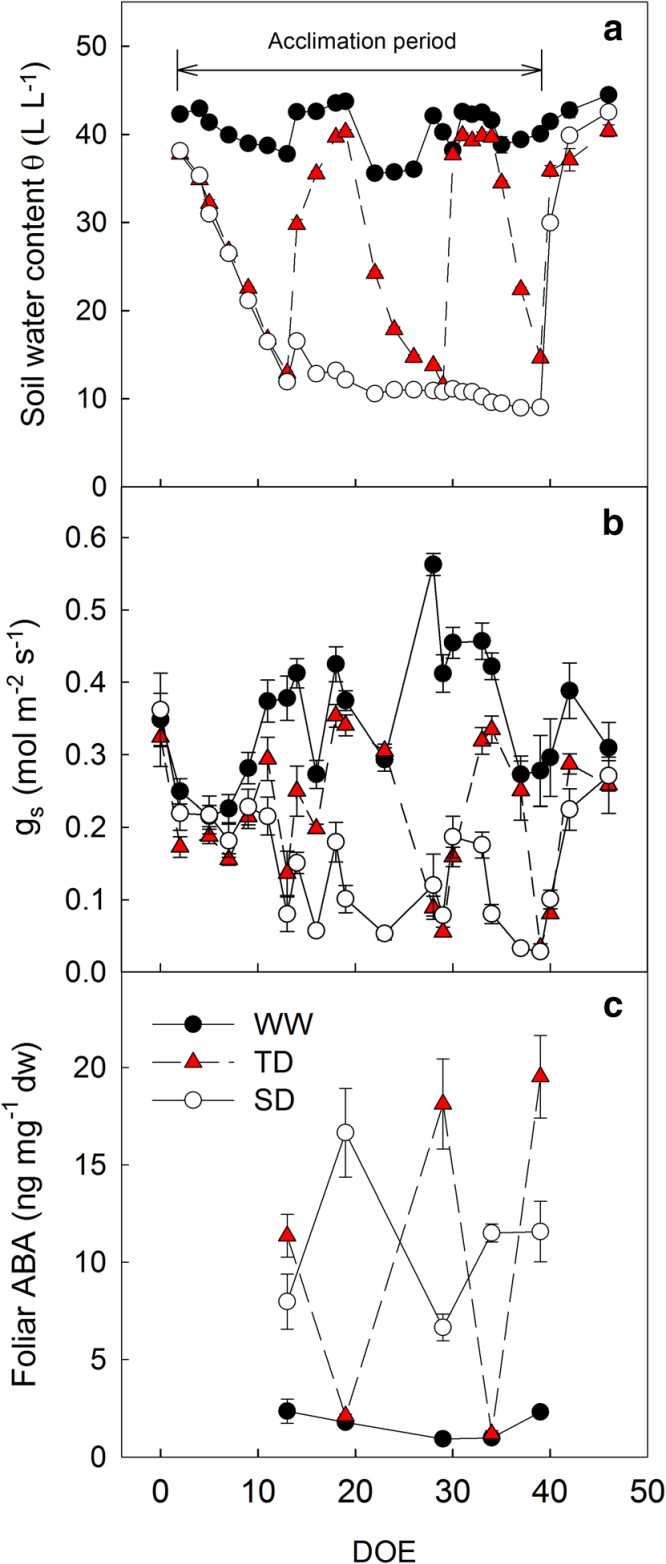
Soil water content (**a**, *θ*), stomatal conductance (**b**, *g*_s_), and foliar abscisic acid (ABA) concentration (**c**) in the well-watered (WW), transient deficit (TD), and sustained deficit (SD) treatments during the acclimation period. Differential irrigation commenced on day 0 of experiment (DOE). Data are mean ± SE, *n* = 6

Irrigation strategies successfully maintained the volumetric soil water content (*θ*) significantly different between treatments (Fig. 2a; Table S1). The *θ* of the well-watered (WW) treatment was maintained above 36% during the entire experiment, while that of the sustained deficit (SD) treatment was lower than 13% starting on the 14th and until the 39th DOE. Interestingly, in order to maintain the desired mid-morning stomatal conductance in the SD vines (0.05 < *g*_s_ < 0.15 mol m^−2^ s^−1^) progressively lower *θ* had to be applied (from 13% on day 14 to 9% on day 39) during the acclimation period, probably due to the vines’ acclimation to low water availability. The transient water deficit treatment (TD) underwent three cycles of dehydration–rehydration in which *θ* fluctuated between 14 and 39% during each cycle (Fig. 2).

In accordance with *θ*, *g*_s_ throughout the acclimation period was higher in the WW vines (0.217–0.563 mol m^−2^ s^−1^), lower in the SD vines (0.187–0.027 mol m^−2^ s^−1^), and fluctuated between 0.034 and 0.341 mol m^−2^ s^−1^ in the TD vines (Fig. 2b; Table S2). On some days, the SD vines exceeded the *g*_s_ values planned for this treatment (*g*_s_ < 0.15 mol m^−2^ s^−1^), reflecting the difficulty to predict the stomatal response to a certain *θ* over time and under different atmospheric conditions. The measured *g*_s_ values suggest that the SD vines experienced moderate stress during most of the acclimation period (*g*_s_ between 0.10 and 0.05 mol m^−2^ s^−1^), with severe stress between the 37th and the 39th day (*g*_s_ < 0.05 mol m^−2^ s^−1^; according to the stress definitions of Medrano et al. 2002). The TD treatment reached minimum *g*_s_ values of 0.137, 0.089, and 0.034 mol m^−2^ s^−1^ on the 13th, 28th and 39th DOE, respectively. After the acclimation period (i.e. after the 39th day of the experiment), all treatments were irrigated as the WW and *g*_s_ increased to non-stressed levels, reaching values of 0.27–0.31 mol m^−2^ s^−1^ (Fig. 2b), which implies that no permanent damage was inflicted by the drought stress.

During the whole acclimation period lower ABA concentration was measured in the WW, higher in the SD and fluctuated in the TD following the *θ* trend. Interestingly, when TD vines were exposed to low *θ*, they exhibited significantly higher ABA concentration than SD, regardless of similar *θ*. However, there was no trace of these high levels following 6 days of rehydration, suggesting that the effect of water stress (Tombesi et al. 2015) was eliminated (Fig. 2c; Table S3).

### Leaf physiological adjustments following acclimation

The differences in irrigation amounts resulted in large differences between the treatments in both leaf area and vine mass (Table 1). After the acclimation, the WW vines had significantly higher (*P* < 0.05) total leaf area (3147 vs. 1206 cm^2^ vine^−1^), fresh weight (117 vs. 36 g vine^−1^), and dry weight (28 vs. 8 g vine^−1^) when compared to the SD treatment. The leaf area and the fresh and dry weights of the TD vines were intermediate between those of the WW and SD vines.

To assess the vines acclimation to low water availability we characterized the difference between the diameter of the xylem vessels, the xylem vulnerability to cavitation, and the modification of the cell characteristics as can be derived from the pressure–volume curve analysis. The exposure to low irrigation resulted in slightly different (*P* > 0.05) osmotic potential at full turgor (*π*_100_; SD = −1.1, TD = −1.07, and WW = −1.02 MPa), and cell modulus of elasticity (*ε*; WW = 6.1; TD = 8.2, SD = 7.2 MPa). The turgor loss point (Ψ_TLP_) was significantly lower (*P* < 0.05) in the vines acclimated under SD coupled with a lower relative water content at turgor loss point (RWC_TLP_) (−1.31 MPa and 88%, respectively) when compared to those acclimated under TD (−1.17 MPa and 91%, respectively) or WW (−1.08 MPa and 92%, respectively) throughout the experiment (Table 2).

**Table 2:** The leaf pressure–volume curve (PV) parameters of well-watered (WW), transient water deficit (TD), and sustained water deficit (SD) vines after the acclimation period: osmotic potential at full turgor (*π*_100_), mean bulk modulus of elasticity (*ε*), water potential at turgor loss point (Ψ_TLP_), and relative water content at turgor loss point (RWC_TLP_)

Treatment	*π*_100_ (MPa)	*ε* (MPa)	Ψ_TLP_ (MPa)	RWC_TLP_ (%)
WW	−1.02 ± 0.03^a^	6.07 ± 0.55^a^	−1.08 ± 0.03^b^	92.06 ± 0.90^a^
TD	−1.07 ± 0.04^a^	8.16 ± 0.78^a^	−1.17 ± 0.04^ab^	90.91 ± 1.24^a^
SD	−1.10 ± 0.05^a^	7.17 ± 0.71^a^	−1.31 ± 0.06^a^	88.21 ± 2.68^a^

Values are mean ± SE (*n* = 9). Different letters within a column indicate significant differences between treatments (*P* < 0.05) by Tukey Honest Significant Difference (HSD) test

**Fig. 3:**
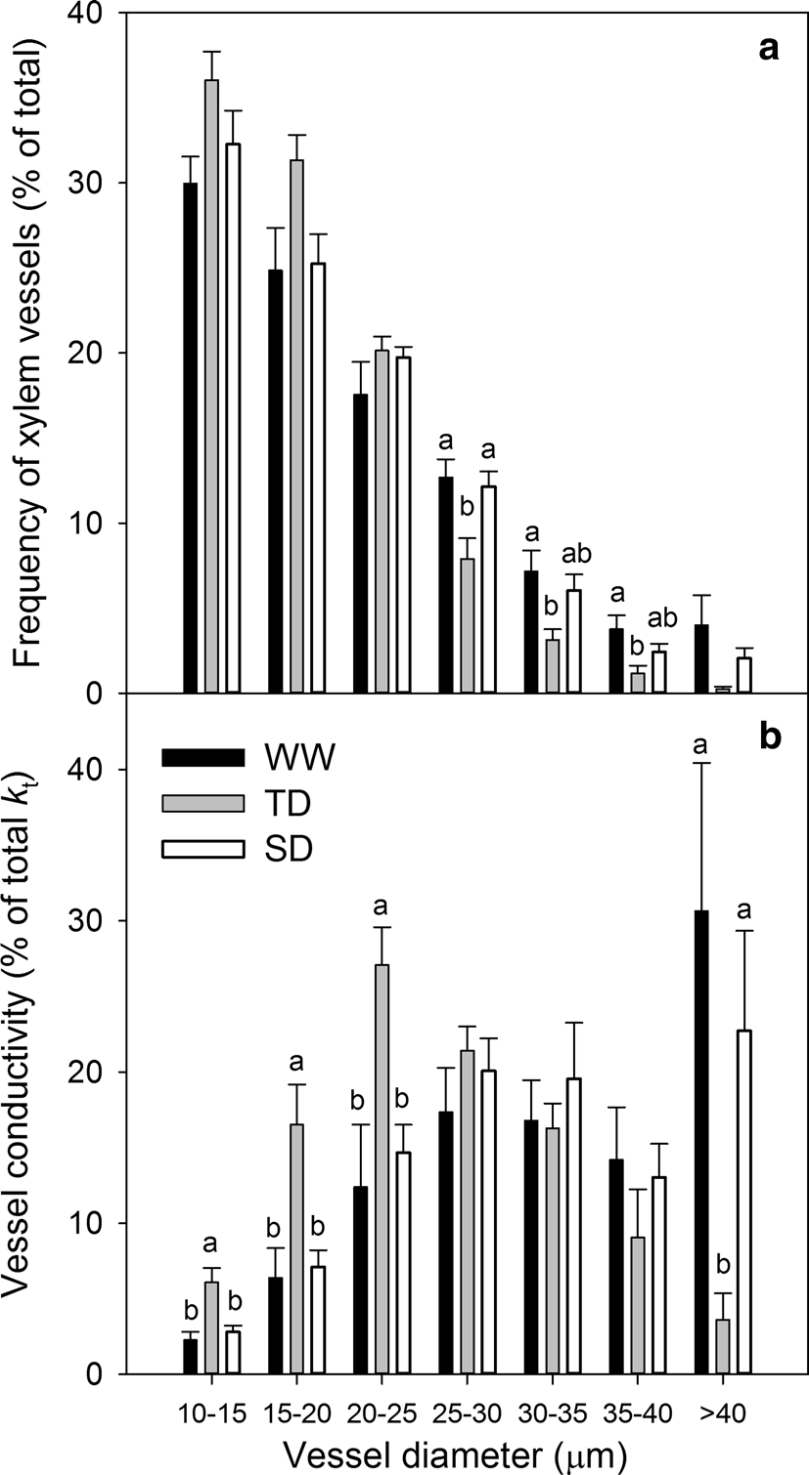
The contribution of different vessel diameter class in petiole cross-section in **a** the relative frequency of vessels number and **b** the relative vessel theoretical conductivity (*k*_t_) in well-watered (WW), transient deficit (TD), and sustained deficit (SD) acclimated vines. Data are mean ± SE, *n* = 7

Measurements of xylem architecture showed that the imposed acclimation treatments resulted in significant differences in xylem differentiation (Table 3; Fig. S2). The WW treatment bore the largest leaf area, petiole cross-section area, average xylem vessel diameter, total xylem area, and theoretical specific hydraulic conductivity (*k*_ts_). While the SD vines had the smallest leaf area (WW = 169; TD = 156; SD = 119 cm^2^), petiole cross-section area (WW = 5.58; TD = 5.47; SD = 4.35 mm^2^), and total xylem vessels area (WW = 0.16; TD = 0.13; SD = 0.12 mm^2^), the TD were the smallest in average xylem vessel diameter (WW = 20.6; TD = 18.3; SD = 20 µm) and *k*_ts_ (WW = 13.82; TD = 8.06; SD = 13.72 mmol m^−1^ s^−1^ MPa). Categorizing xylem vessels based on their size (Fig. 3a) further showed that TD vines had higher proportion of small diameter vessels (10–20 µm) and a significantly lower proportion of high diameter vessels (>25 µm) as compared with the other treatments. On average, less than 5 vessels per petiole were larger than 30 µm in the TD vines, compared with 15 or 10 vessels per petiole in the WW and SD, respectively. Accordingly in the WW vines, much larger proportion of conductance relied on large vessels as compared with the TD (Fig. 3b).

**Table 3:** The petiole xylem architecture of well-watered (WW), transient deficit (TD), or sustained deficit (SD) vines after the acclimation period: the petiole cross-section area, single leaf area (LA), number of bundles, number of xylem vessels, the average xylem vessel diameter (*D*_vessel_), the total vessel area (VA_total_) and the theoretical leaf area specific conductance (*k*_ts_)

Treatment	Petiole area (mm^2^)	LA^*^ (cm^2^)	Bundles (#)	Vessels (#)	*D*_vessel_ (µm)	VA_total_ (mm^2^)	*k*_ts_ (mmol m^−1^ s^−1^ MPa)
WW	5.58 ± 0.29^a^	168.9 ± 2.2^a^	25.7 ± 0.96^a^	424.0 ± 34.1^ab^	20.6 ± 0.65^a^	0.160 ± 0.010^a^	13.82 ± 0.01^a^
TD	5.47 ± 0.30^ab^	155.9 ± 2.5^b^	26.0 ± 1.16^a^	468.3 ± 38.0^a^	18.3 ± 0.41^b^	0.135 ± 0.012^ab^	8.06 ± 0.01^b^
SD	4.33 ± 0.37^b^	119.4 ± 3.6^c^	22.6 ± 1.29^a^	336.3 ± 21.4^b^	20.0 ± 0.28^a^	0.120 ± 0.003^b^	13.72 ± 0.00^a^

Samples were collected only from leaves that developed after the imposition of the acclimation treatments. Values are mean ± SE (*n* = 7). Different letters within a column indicate significant differences between treatments (*P* < 0.05) by Tukey Honest Significant Difference (HSD) test

* LA values presented in Table 1 account also for the small leaves in the plant and thus are significantly smaller than the values below.

**Fig. 4:**
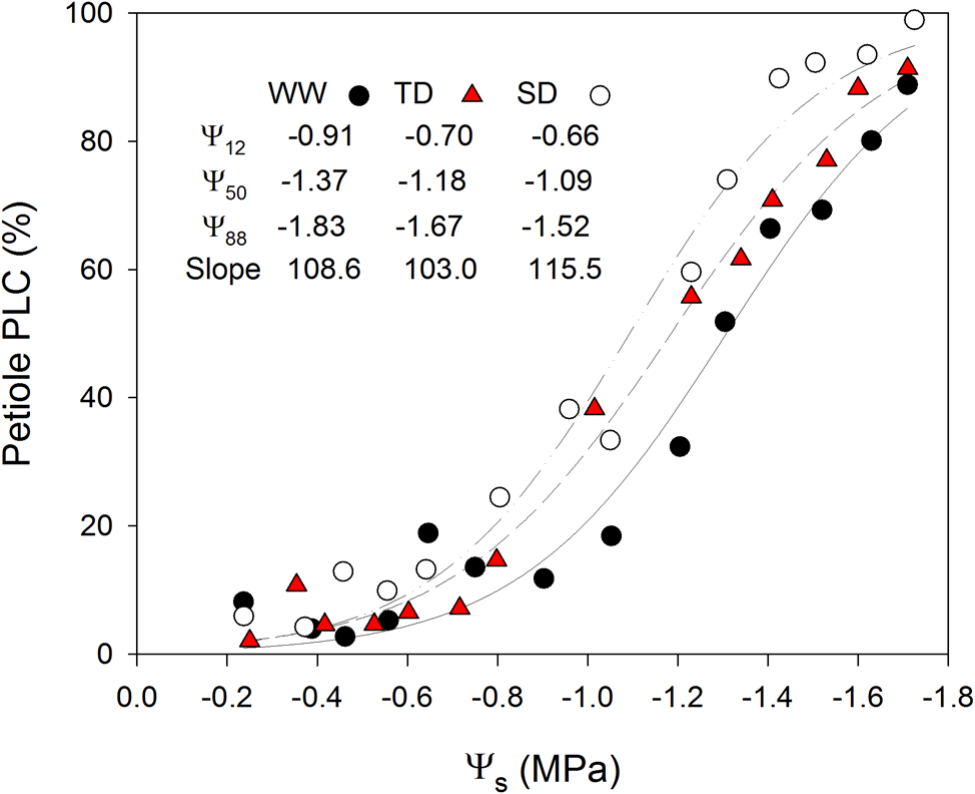
The petiole xylem vulnerability curves (VC) of the well-watered (WW), transient deficit (TD), and sustained deficit (SD) treatments. A sigmoidal regression (3 parameters) between the petiole percent loss of conductivity due to embolism (PLC) and the stem water potential (Ψ_s_). Data are means of three petioles from the same shoot. All VC parameters—stem xylem tension causing 12, 50, or 88% loss of hydraulic conductivity (Ψ_12_, Ψ_50_, Ψ_88_; MPa), and the sigmoidal regression slope (%/MPa) were derived from the sigmoidal regression of the entire data set

The analysis of the xylem vulnerability curves (VC) (Fig. 4) resulted in substantial differences between the treatments, but not as expected. Comparison of the water potential at which half of the conductance is lost (PLC_50 %_) showed that the WW treatment, though bearing the largest vessels (normally coinciding with higher xylem vulnerability), was the least vulnerable to cavitation (PLC_50 %_ = −1.31 MPa). On the other hand, the SD treatment was significantly the most vulnerable to cavitation (PLC_50 %_ = −1.09 MPa). The VC of the TD treatment was intermediate (PLC_50 %_ = −1.18 MPa). A similar range of variance (~0.3 MPa) was also measured for the 12% loss of conductance (PLC_12 %_; WW = −0.91 MPa; TD = 0.7 MPa; SD = −0.6 MPa) and 88% loss of conductance (PLC_88 %_; WW = −1.83 MPa; TD = −1.67 MPa; SD = −1.52 MPa). The treatments had similar slopes (WW = 108%/MPa; TD = 103%/MPa; SD = 116%/MPa) but the linearized model of the VC revealed significant differences between the treatments (*P* = 0.0003) (Fig. S3).

### Hydraulic regulation of drought-acclimated grapevines

**Fig. 5:**
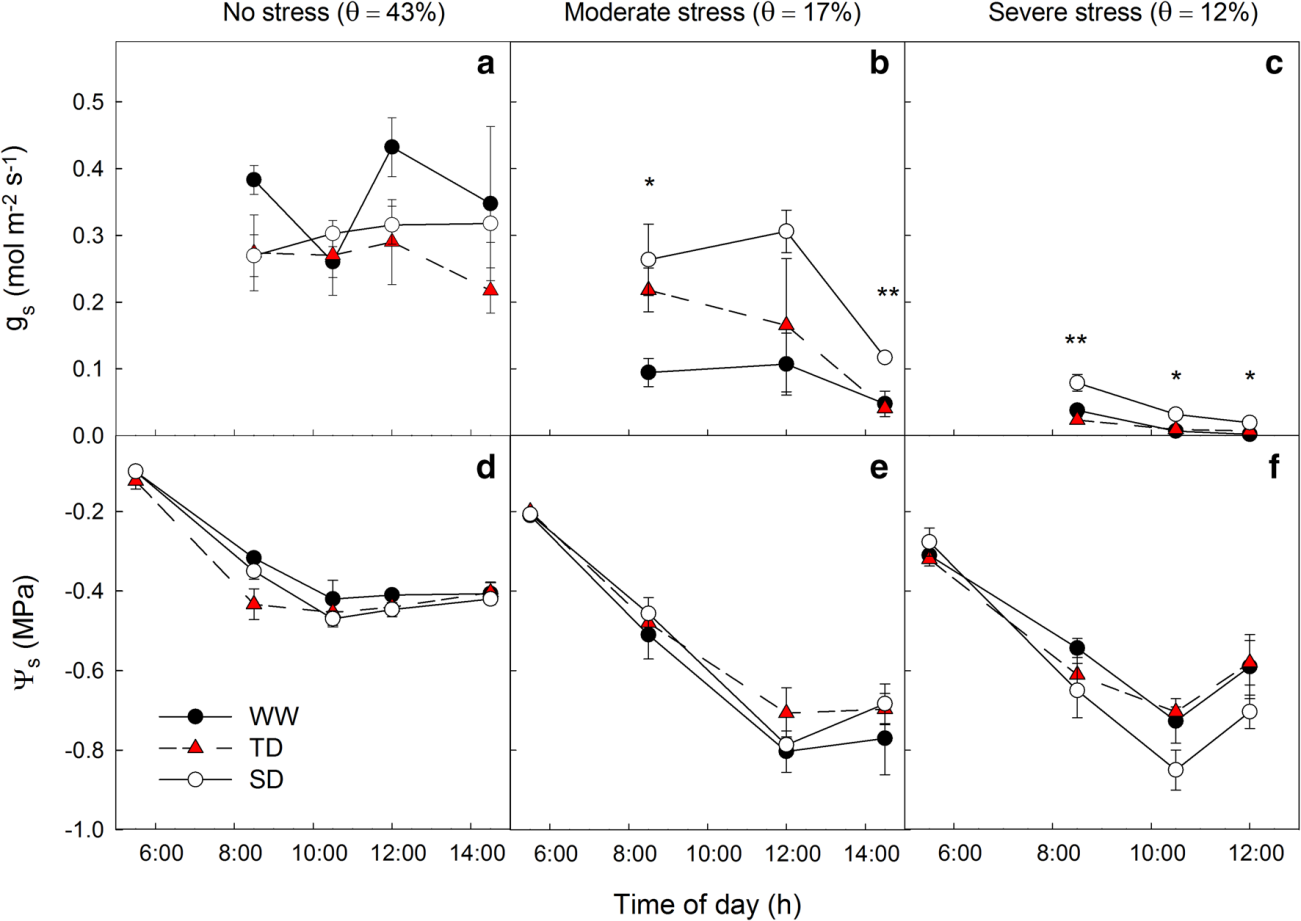
Daily course of the stomatal conductance (**a**–**c**, *g*_s_) and the stem water potential (**d**–**f**, Ψ_s_) of vines acclimated under well-watered (WW), transient deficit (TD), and sustained deficit (SD) condition, when subjected to no-water stress (**a**, **d**), moderate water stress (**b**, **e**), and severe water stress (**c**, **f**) conditions. Following the acclimation period all vines were maintained under full irrigation before subjected to different soil water content (*θ*). Data are mean ± SE, *n* = 3. * or ** represents significant difference (*P* < 0.05 or *P* < 0.01) between the WW and SD treatment

In light of the above-mentioned differences in the physiological characteristics between the treatments, the hydraulic regulation of the acclimated vines was compared in response to different levels of drought. The vines’ water relations were measured over the course of a day at three different levels of *θ* (43, 17 or 12%), presenting clear differences between the treatments. At high soil water availability (*θ* = 43%; Fig. 5a, d), vines from all treatments maintained similar Ψ_s_ (~−0.45 MPa) and *g*_s_ (0.2–0.45 mol m^−2^ s^−1^; Fig. 5a) throughout the day. As soon as the vines were subjected to low soil water content (*θ* = 12 or 17%; Fig. 5b, c, e, f), the SD plants maintained higher *g*_s_ as compared with the TD and WW ones. For example, when *θ* was 17% (Fig. 5b, e), the *g*_s_ in SD vines at midday was significantly higher than in WW vines (0.3 and 0.1 mol m^−2^ s^−1^, respectively) despite similar Ψ_s_ of −0.79 MPa. When *θ* was 12% (Fig. 5c, f), midday *g*_s_ of the SD treatment were at least twice as high as the WW treatment (ranging from 0.009 to 0.034 compared with 0 to 0.004 mol m^−2^ s^−1^ in SD and WW, respectively) in every measurement.

**Fig. 6:**
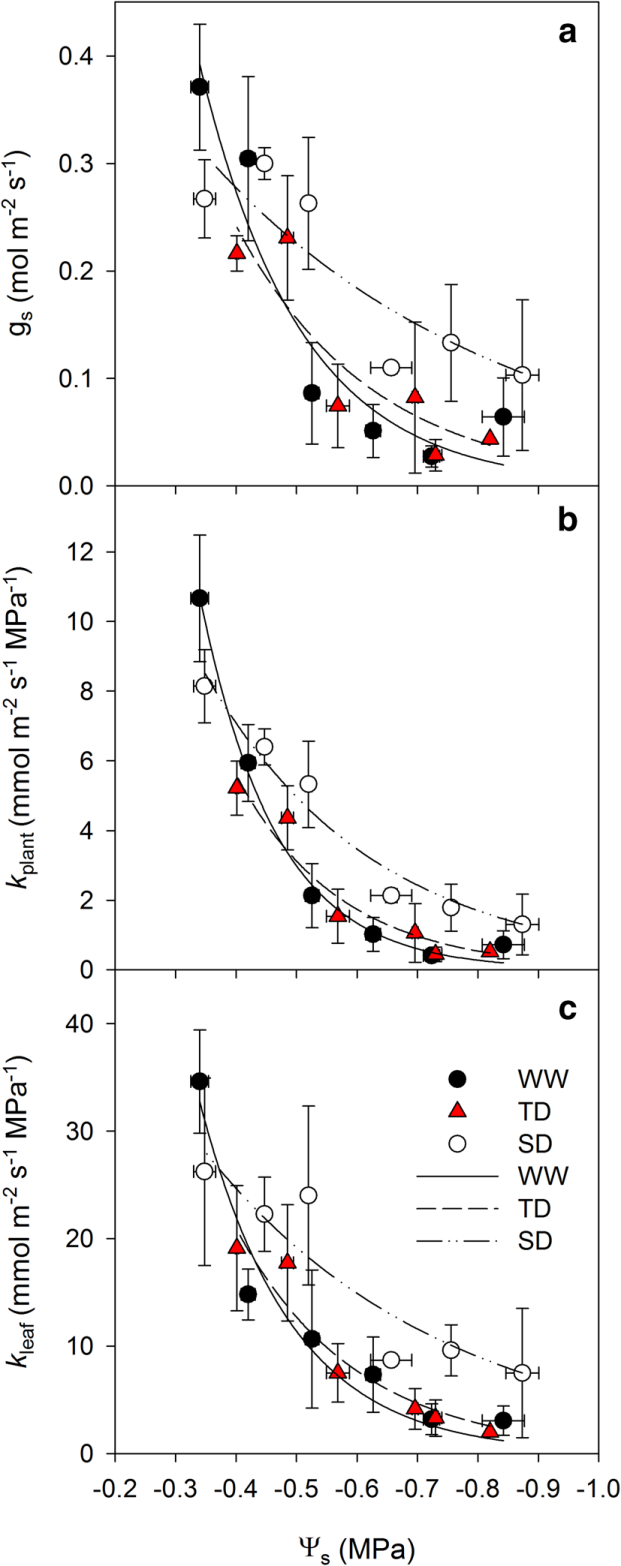
Regression between stem water potential (Ψ_s_) and stomatal conductance (**a**, *g*_s_), the whole plant conductance (**b**, *k*_plant_ specific to leaf area), and the leaf conductance (**c**, *k*_leaf_ specific to leaf area) of the well-watered (WW), transient deficit (TD), and sustained deficit (SD) treatments. Data were taken from the 3 days presented in Fig. 5 and averaged based on similar values of Ψ_s_. Each point is the mean ± SE, *n* = 3–7

**Fig. 7:**
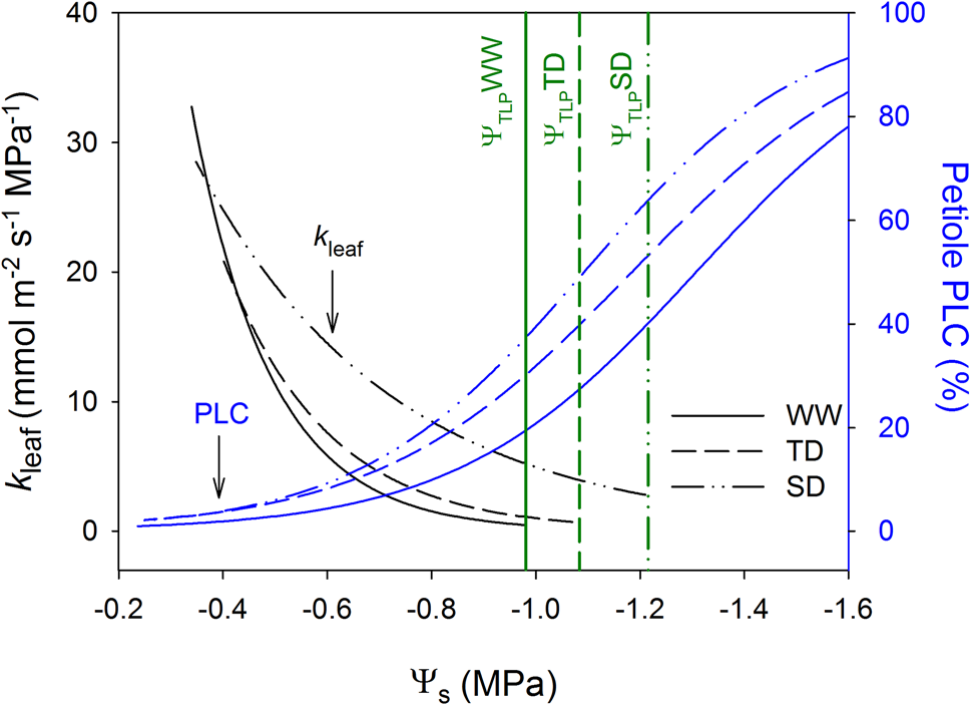
Embolism in the petiole is not the primary cause for the reduction in leaf conductance. Regression between the stem water potential (Ψ_s_) and leaf conductance (*k*_leaf_ specific to leaf area) or the percent loss of conductivity due to embolism in the petiole (PLC) of the well-watered (WW), transient water deficit (TD), and sustained water deficit (SD) acclimated vines. Sigmoidal regression of petiole PLC is reproduced from Fig. 4. *k*_leaf_ regression is reproduced from Fig. 6c. The turgor loss point (Ψ_TLP_) of WW, TD, and SD was modified to Ψ_s_ (from Ψ_l_) based on the average difference between Ψ_s_ and Ψ_l_ (0.1 MPa during the three full-day measurements) and plotted as *vertical lines*

When comparing the regressions of *k*_plant_, *k*_leaf_ or *g*_s_ against Ψ_s_, the SD treatment presented higher values for all of these parameters as compared with the WW and TD treatments (Fig. 6; statistical comparison at Table S4) at Ψ_s_ lower than −0.5 MPa. As Ψ_s_ became more negative, *g*_s_, *k*_leaf_ and *k*_plant_ were reduced, regardless of the acclimation treatment. The *g*_s_ and *k*_plant_ reduction was sharper in the WW and TD as compared with SD as Ψ_s_ became more negative, presenting the modification of the vines’ water relations in respect to the irrigation treatment during the acclimation period. However, comparison of petiole embolism and *k*_leaf_ reduction showed that the large majority of *k*_leaf_ reduction in all treatments (from above 30 to below 10 mmol m^−2^ s^−1^ MPa^−1^) occurred at Ψ_s_ higher than −0.6 MPa, equivalent only to less than 20% PLC (Fig. 7). The difference between 50% *k*_leaf_ reduction and 50% PLC was at least 0.4 MPa (in SD) and up to 0.8 MPa (in WW). Even more so, the SD vines were more vulnerable to cavitation than the WW ones, but showed milder reduction in *k*_leaf_ as Ψ_s_ became more negative. On the other hand, the *k*_leaf_ decline of each treatment could be associated with their Ψ_TLP_ (~0.2 MPa difference in Ψ_TLP_ and *k*_leaf_ 50% between the WW and SD) and explain the sharper *k*_leaf_ reduction of the WW treatment (Fig. 7).

## Discussion

The results support the hypotheses that acclimation to water deficit modifies both the grapevine hydraulic properties and the regulation of gas exchange in future drought events; drought acclimation resulted in morphological modifications of the hydraulic system in grapevines, which in turn allowed the vines to maintain higher *g*_s_ and *k*_plant_ (compared to vines acclimated to high water availability) under drought conditions. In agreement with previous research (Patakas et al. 1997; Martorell et al. 2014), the vines acclimated to low soil water content had a lower leaf area (LA) and Ψ_TLP_ than vines acclimated to well-watered conditions. These modifications induced by the acclimation possibly contributed, in the subsequent drought event, to the maintenance of higher rates of gas exchange while going to lower Ψ_s_. Similar results were observed in rice (Gupta et al. 1989) and sunflowers (Matthews et al. 1984), where acclimation to water deficit resulted in osmotic adjustment, which in turn lead to higher *g*_s_ and growth in a subsequent drought event. The accumulation of solutes under water deficit conditions is well documented in grapevine (Patakas et al. 2002; Hochberg et al. 2013), and the consequential reduction of Ψ_TLP_ (Table 2) can explain the maintenance of improved leaf turgor, leading to higher stomatal conductance (Franks and Farquhar 1998). The modification of *g*_s_ ~ Ψ_s_ relation is particularly interesting when considering its high variability in grapevines, suggesting that in addition to the soil type and the scion-rootstock interaction (Lavoie-Lamoureux et al. 2017), also the acclimation conditions should be considered.

Growth inhibition, expressed by the large size differences between treatments (as leaf area or weight; Table 1), is one of the first and most noticeable responses to water limitation as it allows plants to conserve resources (Lebon et al. 2006). Size differences could have created several hydraulics or non-hydraulic effect on gas exchange variability between treatments. Since transpiration is largely affected by the leaf area, but water supply was limited by the pot size (that was uniform across treatments), the demand/supply ratio was significantly larger in the WW treatment, possibly leading to tighter down-regulation of *g*_s_ under stress conditions. To mitigate the differences in demand/supply ratio, we applied frequent irrigation (every 2 h) that maintained a constant *θ* in the moderate and severe stress days (Fig. 5), thus diminishing the pot volume effect (Ray and Sinclair 1998). However, other authors suggested that it is the actual root length density (and not water availability), that affects stomatal closure (Shein and Pachepsky 1995). Moreover, other internal factor involving size such as sink demand, leaf sugar level, and starch storage were also shown to affect *g*_s_ (Goldschmidt and Huber 1992). These factors weren’t the focus of this study, but should be further investigated to fully understand drought acclimation.

The comparison of the two acclimation treatments (TD and SD) showed that the difference in the acclimation processes is more than just a function of the drought integral (sum of water stress period). For example, when the TD treated vines were exposed to minimum *θ*, ABA concentration was double the amount in TD than in SD vines. The difference in ABA concentration suggests that on a molecular level drought response was higher for the TD vines, an effect possibly correlated with the Ψ_TLP_ differences between the treatments (McAdam and Brodribb 2016). On the same note, the average xylem vessel area and *k*_ts_ in the TD vines were significantly smaller than in SD vines. These differences could be attributed to the alteration of many regulatory and developmental processes that take effect shortly after drought is applied (Cramer et al. 2013). Accordingly, molecular regulation could continue shaping the development of well-watered plants in accordance with a previous water stress, possibly with epigenetics as the base for the stress “memory” (Chinnusamy and Zhu 2009). Under such conditions, the higher assimilation would also increase the resources that could be invested in the acclimation process, thus giving rise to the hypothesis that it would lead to larger modifications during the acclimation period. That said, many other acclimation processes, such as leaf area reduction and osmotic adjustment, underwent significantly larger modifications in the SD treatment than in TD, demonstrating the complexity of the acclimation process.

Remarkably, our results showed 4–8 bars difference between 50% *k*_leaf_ or *g*_s_ and 50% PLC (Fig. 7), challenging the commonly assumed involvement of embolism in the fast down-regulation of *g*_s_ (Zufferey et al. 2011) and *k*_leaf_ (Sack and Holbrook 2006). In agreement, computed tomography of intact petioles showed very little embolism (<25%) when *k*_leaf_ (measured as in the current study) was completely down-regulated (Charrier et al. 2016). These findings reinforce the importance of other (than embolism) mechanisms in the down-regulation of *k*_leaf_: deformation of conduits under tension (Zhang et al. 2014, 2016), modification of the hydraulic properties of the extra-vascular compartment (Trifiló et al. 2016), or molecular regulation of membrane permeability through the interaction between aquaporins and ABA (Vitali et al. 2016), provide plausible explanations for the fast reduction in *k*_leaf_ under water stress. ABA was recently shown to peak when approaching the Ψ_TLP_ (McAdam and Brodribb 2016) and also to affect aquaporin-related genes expression and the *k*_leaf_ (Vitali et al. 2016), thus explaining how the lower Ψ_TLP_ of the SD vines led to higher *k*_leaf_ under similar Ψ_s_ when compared to the WW treatment (illustrated in Fig. 6). Alternatively, leaf shrinkage, mediated through turgor pressure, has also been suggested to play a key role in the *k*_leaf_ decline (Scoffoni et al. 2014). Although the mechanical nature of the interaction between turgor pressure and *k*_leaf_ is not clear, it gains support from several other comparisons across grapevine cultivars, which showed that genotypes with lower Ψ_TLP_ tend to maintain higher *g*_s_ and *k*_leaf_ (During and Loveys 1982; Tombesi et al. 2014; Martorell et al. 2014, 2015).

The larger vulnerability to cavitation in combination with the highest *g*_s_ and lowest Ψ_s_ observed in the SD vines, challenge previous suggestion of stomatal regulation through petiole cavitation (Zufferey et al. 2011). Rather, the combination implies that the majority of *g*_s_ down-regulation takes place before substantial limitation is caused by embolism in the petiole. Accordingly, our previous research demonstrated that the majority of *g*_s_ decline (to less than 20% of the optimal conditions) occurs at Ψ_s_ values higher than those leading to embolism in the leaf veins, petioles or shoots (Hochberg et al. 2016a, b). Similarly, in the current experiment *k*_leaf_ and *g*_s_ were mutually down-regulated as Ψ_s_ became more negative, but in agreement with our previous publications, no petiole embolism was measured under such Ψ_s_ (Fig. 7). These results undermine the possibility that petiole cavitation controls stomatal regulation (Nardini and Salleo 2000) and strongly support the hypothesis that grapevines have evolved a strategy of cavitation avoidance, rather than one of cavitation tolerance with diurnal cycles of embolism repair (Hochberg et al. 2016b). It is important to mention that the correlation between xylem cavitation and stomatal regulation has been shown in several multi-species analyses (Cruiziat et al. 2002; Brodribb et al. 2003; Manzoni 2014). Our results do not contradict these finding, rather they suggest that the coordination between *g*_s_ and the petiole vulnerability to cavitation was shaped by evolution and is not a direct control mechanism.

Differently from our original hypothesis, vines that were acclimated to water deficit did not show improved resistance to cavitation (Fig. 4) although xylem architecture was modified. The literature suggests that smaller vessels, when compared to larger vessels in the xylem tissue of the same plant, normally exhibit improved vulnerability to cavitation (Tyree et al. 1994; Guet et al. 2015). It has also been found that exposure of hybrid poplars to drought resulted in both smaller vessel and improved cavitation resistance (Plavcova and Hacke 2012). In the current experiment, water deprivation led to smaller leaves with smaller xylem area, bearing smaller vessels (Table 3; Fig. 3a), as was previously reported for grapes (Lovisolo and Schubert 1998; Hochberg et al. 2015). The higher proportion of large vessels, which in turn contribute largely to the higher *k*_t_ in the WW treatment (Fig. 3b), should imply a lower resistance to cavitation. However, the vines acclimated to drought showed lower resistance to cavitation than the WW treatment, despite the acclimation effect on their morphological and anatomical traits.

These discrepancies could have arisen from two main causes: (1) the measured petioles were at different stages of development; and (2) the SD petioles suffered from cavitation fatigue. The first potential cause is reasonable, considering that during development the xylem has the potential to have significantly differing VC (Plavcova and Hacke 2012) and that the SD plants slowed development and growth (Table 1). The measured petioles were chosen before the treatments were applied, meaning that they had the same age (in days). However, reduced growth rate could have resulted in developmental changes (despite similar mature leaf appearance for all sampled leaves) that are known to impact xylem vulneravility to cavitation: alteration of the primary/secondary xylem ratio, lignin deposition, and/or pit pectin content (Herbette et al. 2015).

Alternatively, cavitation fatigue (i.e. the phenomenon of increased cavitation vulnerability in response to cavitation/refilling cycles; Hacke et al. 2001) could have increased the xylem vulnerability of the SD treatment. As the SD and TD vines were exposed to stress during the acclimation period, it is possible that some of their vessels were embolized and later refilled following rehydration. However, our VCs were constructed 3 weeks after rehydration, and the possibility of cavitation fatigue modifying VCs over times scales of this length is still in question. Stiller and Sperry (2002) suggested that the effect of cavitation fatigue is reduced as a function of the number of days since refilling, showing that in sunflowers it was completely reversible 4 days after irrigation. Conversely, other authors (Christensen-Dalsgaard and Tyree 2014; Schreiber et al. 2015) found that cavitation fatigue was still effective months after the cavitation event. Hacke et al. (2001) showed that the phenomenon is species dependent, implying that the discrepancy could be genetic. However, as neither the mechanism, nor the conditions, in which cavitation fatigue is inflicted or reversed are known, it is hard to determine if this was the cause for the larger vulnerability of the SD vines.

## Conclusion

Our results show that grapevine acclimation to water deficit modifies the xylem architecture, Ψ_TLP_, and VCs. Some of the findings contradicted our original hypothesis and challenge several conceptions regarding plant hydraulics. For one, acclimation to water stress and differentiation of smaller vessel area in petioles did not lead to improved xylem resistance to cavitation. Additionally, petiole embolism cannot account for the reduction of *k*_leaf_ and *g*_s_ under water stress. The interaction between Ψ_TLP_ and *k*_leaf_, along with its effect on the plant’s ability to maintain adequate gas exchange under stress should be further explored.

### *Author contribution statement*

UH and JCH conceived, designed and executed the research with the help of AGB. RDS performed microscopy analysis of petioles. AD and AF sampled and performed ABA analysis. HC assisted with hydraulic measurements. EP coordinated all the experiment activities. UH and JCH analyzed the data and wrote the manuscript. All authors discussed the results and revised the manuscript.

## Electronic supplementary material

Below is the link to the electronic supplementary material.

**Fig. S1** Daily air temperature (°C) and vapor pressure deficit (VPD, kPa) in the greenhouse during the course of the acclimation period (PDF 181 kb)

**Fig. S2** Petiole cross-section microscopy images from well-watered (WW), transient water deficit (TD), and sustained water deficit (SD) acclimated vines (PDF 358 kb)

**Fig. S3** Statistical comparison of the xylem vulnerability curves from the three acclimation treatments (PDF 340 kb)

**Table S1** Soil water content (*θ*, %) in the WW, TD, and SD pots during the acclimation period (Fig. 2a) (PDF 144 kb)

**Table S2** Stomatal conductance (g_s_, H_2_O m^−2^ s^−1^) in the WW, TD, and SD pots during the acclimation period (Fig. 2b) (PDF 98 kb)

**Table S3** Abscisic acid (ABA) concentration (ng mg^−1^ dw) in WW, TD, and SD leaves during the acclimation period (Fig. 2c) (PDF 86 kb)

**Table S4** Average leaf (*k*_leaf_), plant (*k*_plant_), and stomatal (g_s_) conductance at given classes of stem water potential (Ψ_s_). Values are from the post-acclimation 3-day drought experiment represented in Fig. 6 (PDF 196 kb)

## Abbreviations

DOE: Day after the initiation of the irrigation experiment
*g*_s_: Stomatal conductance
*k*_leaf_: Leaf hydraulic conductance specific to leaf area
*k*_plant_: Whole plant hydraulic conductance specific to leaf area
*k*_t_: Petiole theoretical hydraulic conductivity
*k*_ts_: Petiole theoretical specific hydraulic conductivity
PLC: Percentage loss of conductivity
SD: Sustained water deficit
TD: Transient water deficit
VC: Xylem vulnerability curve
WW: Well-watered
*θ*: Soil water content
Ψ: Water potential (_l, leaf; s, stem_)
Ψ_TLP_: Turgor loss point
