# Supplementary material for: Grapevine acclimation to water deficit: the adjustment of stomatal and hydraulic conductance differs from petiole embolism vulnerability
Source: Planta. 2017 Feb 18;245(6):1091–104. doi: 10.1007/s00425-017-2662-3 (PMC5432590; doi:10.1007/s00425-017-2662-3)
Supplement: Supplementary file 1 — Fig. S1 Daily air temperature (°C) and vapor pressure deficit (VPD, kPa) in the greenhouse during the course of the acclimation period (PDF 181 kb) [file 425_2017_2662_MOESM1_ESM.pdf]

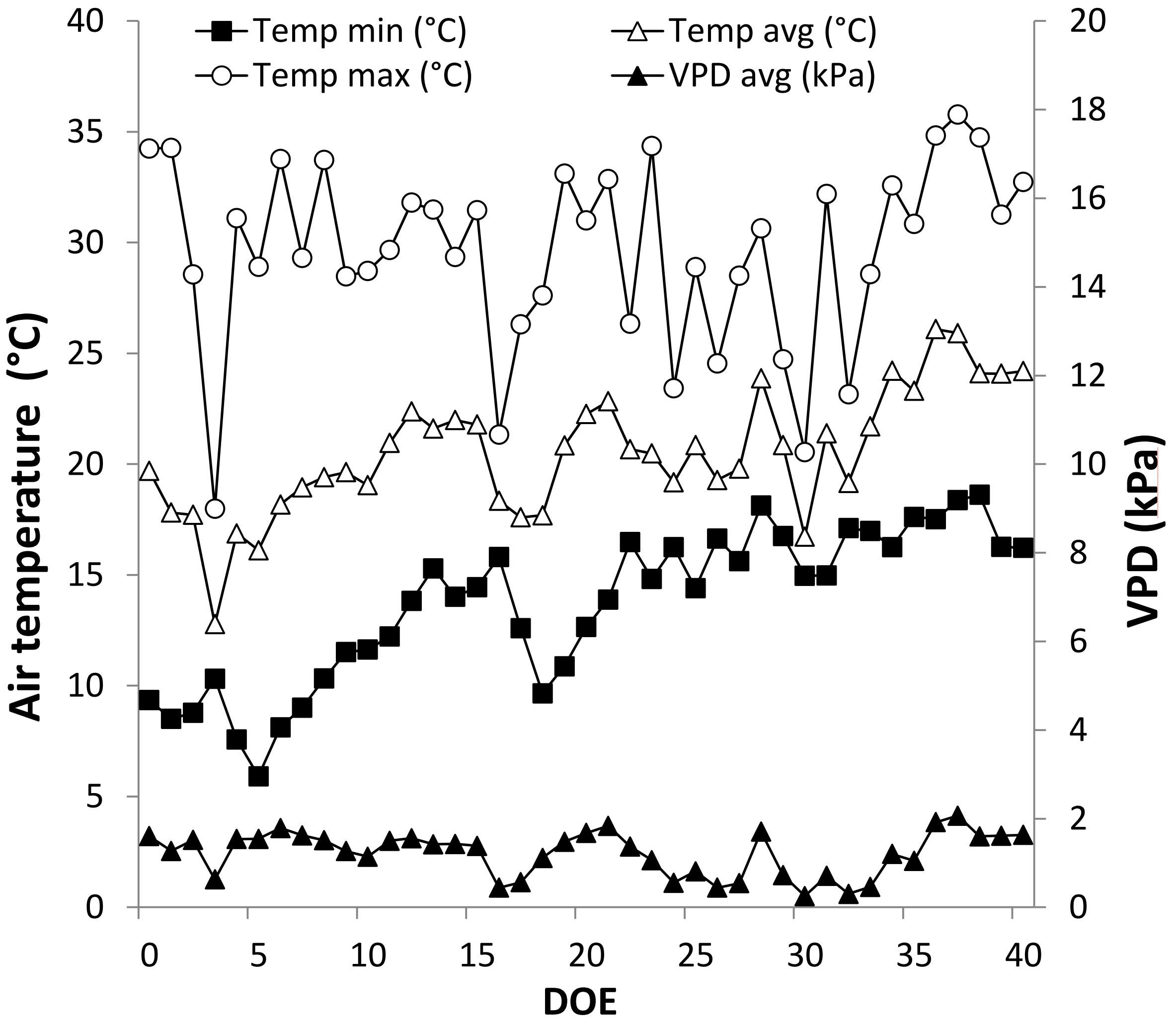

Fig. S1 Daily air temperature (°C) and vapor pressure deficit (VPD, kPa) in the greenhouse during the course of the acclimation period
