# Supplementary material for: Grapevine acclimation to water deficit: the adjustment of stomatal and hydraulic conductance differs from petiole embolism vulnerability
Source: Planta. 2017 Feb 18;245(6):1091–104. doi: 10.1007/s00425-017-2662-3 (PMC5432590; doi:10.1007/s00425-017-2662-3)
Supplement: Supplementary file 2 — Fig. S2 Petiole cross-section microscopy images from well-watered (WW), transient water deficit (TD), and sustained water deficit (SD) acclimated vines (PDF 358 kb) [file 425_2017_2662_MOESM2_ESM.pdf]

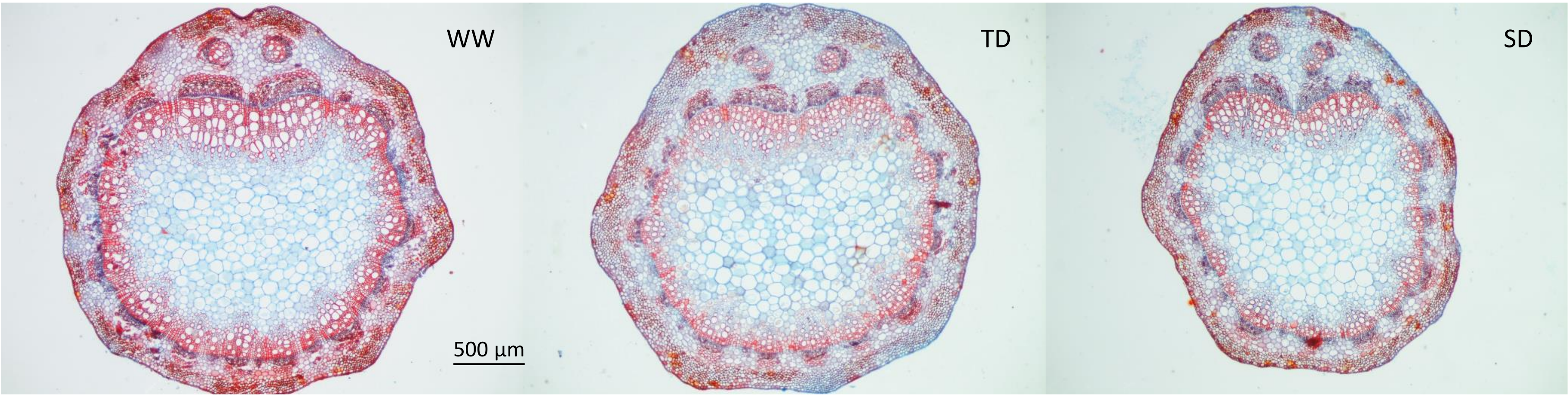

Fig. S2 Petiole cross-section microscopy images from grapevines acclimated to well-watered (WW), transient water deficit (TD) and sustained water deficit (SD)
