# Supplementary material for: Grapevine acclimation to water deficit: the adjustment of stomatal and hydraulic conductance differs from petiole embolism vulnerability
Source: Planta. 2017 Feb 18;245(6):1091–104. doi: 10.1007/s00425-017-2662-3 (PMC5432590; doi:10.1007/s00425-017-2662-3)
Supplement: Supplementary file 3 — Fig. S3 Statistical comparison of the xylem vulnerability curves from the three acclimation treatments (PDF 340 kb) [file 425_2017_2662_MOESM3_ESM.pdf]

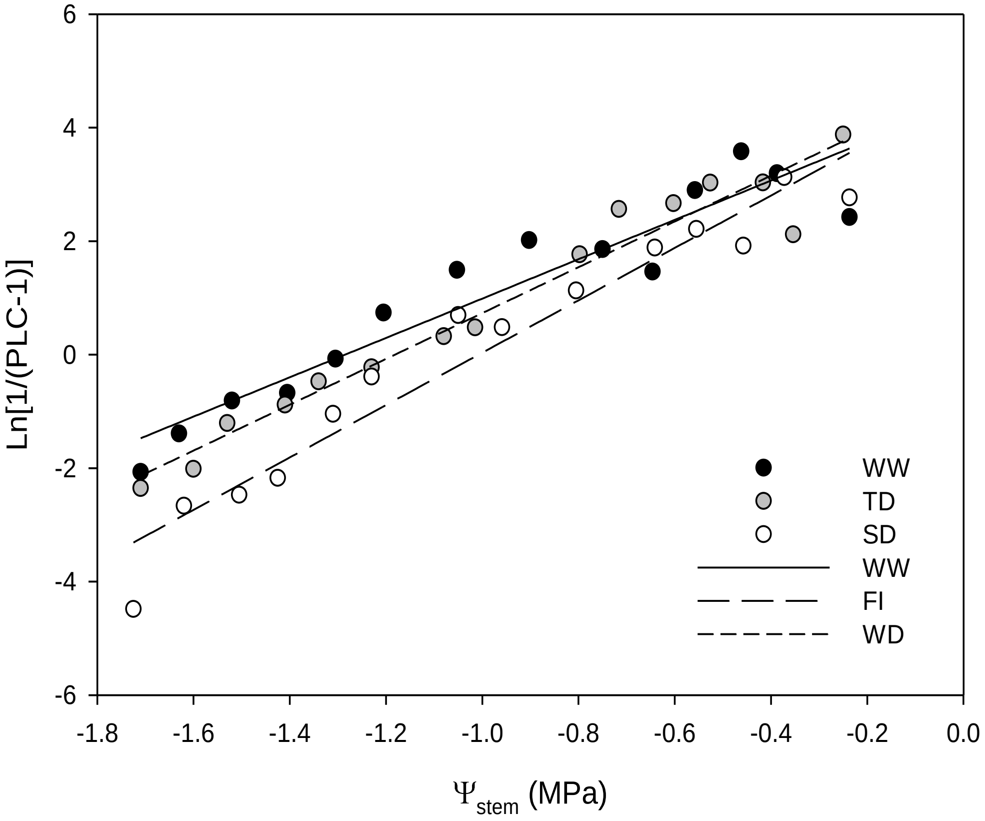

Effect Tests					
**Source**	**Nparm**	**DF**	**Sum of Squares**	**F Ratio**	**Prob>F**
Ψs	1	1	155.91356	469.1518	<0.0001
Treatment	2	2	6.67009	10.0353	0.0003

Fig S3 Statistical comparison of the xylem vulnerability curves from the three acclimation treatments. The sigmoidal curves of well watered (WW), transient deficit (TD), and sustained deficit (SD) treatments from Fig. 3 were linearized and compared using a regression model with Ψ_s and the treatment as fixed model effects (P<0.05)
