# Supplementary material for: Grapevine acclimation to water deficit: the adjustment of stomatal and hydraulic conductance differs from petiole embolism vulnerability
Source: Planta. 2017 Feb 18;245(6):1091–104. doi: 10.1007/s00425-017-2662-3 (PMC5432590; doi:10.1007/s00425-017-2662-3)
Supplement: Supplementary file 4 — Table S1 Soil water content (θ, %) in the WW, TD, and SD pots during the acclimation period (Fig. 2a) (PDF 144 kb) [file 425_2017_2662_MOESM4_ESM.pdf]

Table S1 Soil water content (θ, %) in the WW, TD, and SD pots during the acclimation period (Fig. 2a). Different letters for each row (DOE= Days of Experiment) denotes significant differences (*P*<0.05) between treatments as tested by Tukey HSD

DOE	WW		TD		SD	
2	42.29	a	37.76	b	38.10	b
4	42.93	a	34.86	b	35.32	b
5	41.35	a	32.17	b	30.95	b
7	39.93	a	26.76	b	26.48	b
9	38.95	a	22.52	b	21.14	b
11	38.71	a	16.76	b	16.48	b
13	37.76	a	12.90	b	11.90	b
14	42.52	a	29.76	b	16.52	c
16	42.57	a	35.52	b	12.81	c
18	43.52	a	39.67	a	13.19	b
19	43.71	a	40.24	a	12.14	b
22	35.57	a	24.24	b	10.57	c
24	35.71	a	17.86	b	11.00	c
26	36.00	a	14.71	b	11.00	c
28	42.10	a	13.76	b	10.90	b
29	40.24	a	11.52	b	10.76	b
30	38.19	a	37.67	a	11.10	b
31	42.55	a	39.86	a	10.76	b
32	42.26	a	39.24	a	10.76	b
33	42.48	a	39.81	a	10.24	b
34	41.57	a	39.67	a	9.57	b
35	38.81	a	34.48	b	9.48	c
37	39.38	a	22.38	b	8.95	c
39	40.05	a	14.62	b	9.00	c
40	41.43	a	35.86	b	29.95	c
42	42.71	a	37.10	b	39.86	b
46	44.46	a	40.38	a	42.51	a
