# Supplementary material for: Grapevine acclimation to water deficit: the adjustment of stomatal and hydraulic conductance differs from petiole embolism vulnerability
Source: Planta. 2017 Feb 18;245(6):1091–104. doi: 10.1007/s00425-017-2662-3 (PMC5432590; doi:10.1007/s00425-017-2662-3)
Supplement: Supplementary file 5 — Table S2 Stomatal conductance (gs, H2O m−2 s−1) in the WW, TD, and SD pots during the acclimation period (Fig. 2b) (PDF 98 kb) [file 425_2017_2662_MOESM5_ESM.pdf]

Table S2 Stomatal conductance (g_s, H_2O mol m^{-2} s^{-1}) in the WW, TD, and SD vines during the acclimation period (Fig. 2b). Different letters for each row (DOE = Days of Experiment) denotes significant differences (*P*<0.05) between treatments as tested by Tukey HSD

**DOE**	**WW**		**TD**		**SD**	
0	0.349	a	0.324	a	0.362	a
2	0.249	a	0.173	a	0.219	a
5	0.216	a	0.188	a	0.217	a
7	0.226	a	0.156	b	0.182	ab
9	0.281	a	0.215	a	0.228	a
11	0.374	a	0.294	ab	0.215	b
13	0.378	a	0.137	b	0.080	c
14	0.413	a	0.250	b	0.151	c
16	0.273	a	0.198	b	0.057	c
18	0.425	a	0.354	b	0.180	c
19	0.375	a	0.341	a	0.101	b
23	0.293	a	0.305	a	0.053	b
28	0.563	a	0.089	b	0.120	b
29	0.412	a	0.056	b	0.079	b
30	0.455	a	0.159	b	0.187	b
33	0.457	a	0.319	a	0.176	b
34	0.422	a	0.335	b	0.080	c
37	0.272	a	0.250	a	0.033	b
39	0.278	a	0.034	b	0.028	b
40	0.296	a	0.081	b	0.101	b
42	0.388	a	0.287	b	0.225	b
46	0.309	a	0.258	a	0.271	a
