# Supplementary material for: Grapevine acclimation to water deficit: the adjustment of stomatal and hydraulic conductance differs from petiole embolism vulnerability
Source: Planta. 2017 Feb 18;245(6):1091–104. doi: 10.1007/s00425-017-2662-3 (PMC5432590; doi:10.1007/s00425-017-2662-3)
Supplement: Supplementary file 6 — Table S3 Abscisic acid (ABA) concentration (ng mg−1 dw) in WW, TD, and SD leaves during the acclimation period (Fig. 2c) (PDF 86 kb) [file 425_2017_2662_MOESM6_ESM.pdf]

Table S3 Abscisic acid (ABA) concentration (ng mg^{-1} DW) in WW, TD, and SD leaves during the acclimation period (Fig. 2c). Different letters for each row (DOE = Days of Experiment) denotes significant differences (*P*<0.05) between treatments as tested by Tukey HSD. DW = Dry weight

DOE	WW		TD		SD	
13	2.35	b	11.35	a	7.98	a
19	1.78	b	2.10	b	16.65	a
29	0.93	c	18.13	a	6.65	b
34	0.98	b	1.18	b	11.50	a
39	2.30	c	19.53	a	11.58	b
