# Supplementary material for: Grapevine acclimation to water deficit: the adjustment of stomatal and hydraulic conductance differs from petiole embolism vulnerability
Source: Planta. 2017 Feb 18;245(6):1091–104. doi: 10.1007/s00425-017-2662-3 (PMC5432590; doi:10.1007/s00425-017-2662-3)
Supplement: Supplementary file 7 — Table S4 Average leaf (k leaf), plant (k plant), and stomatal (gs) conductance at given classes of stem water potential (Ψs). Values are from the post-acclimation 3-day drought experiment represented in Fig. 6 (PDF 196 kb) [file 425_2017_2662_MOESM7_ESM.pdf]

**Table S4** Average leaf ($k_{leaf}$), plant ($k_{plant}$), and stomatal ($g_s$) conductance at given classes of stem water potential ($\Psi_s$). Values are from the post-acclimation three-day drought experiment. Data is presented as averages ± SE (*n*=5-8). Within a column and for each Ψ threshold, different letters identify significant differences between treatments (*P*<0.05) as tested by Tukey-HSD

| | **Ψs (MPa)** | $k_{leaf}$ **(mmol m$^{-2}$ s$^{-1}$ MPa$^{-1}$)** | $k_{plant}$ **(mmol m$^{-2}$ s$^{-1}$ MPa$^{-1}$)** | $g_s$ **(mol m$^{-2}$ s$^{-1}$)** |
|---|---|---|---|---|
| Ψ > -0.5 MPa | | | | |
| WW | -0.39 ± 0.01 a | 22.23 ± 6.24 a | 7.54 ± 1.26 a | 0.31 ± 0.05 a |
| TD | -0.42 ± 0.01 a | 25.20 ± 8.88 a | 5.62 ± 0.70 a | 0.25 ± 0.02 a |
| SD | -0.41 ± 0.02 a | 23.70 ± 3.63 a | 7.12 ± 0.57 a | 0.29 ± 0.02 a |
| -0.7 < Ψ < -0.5 MPa | | | | |
| WW | -0.55 ± 0.02 a | 6.62 ± 3.17 b | 2.00 ± 0.38 b | 0.08 ± 0.02 b |
| TD | -0.59 ± 0.02 a | 11.18 ± 8.55 ab | 2.42 ± 0.75 b | 0.12 ± 0.04 ab |
| SD | -0.58 ± 0.03 a | 17.43 ± 5.42 a | 3.96 ± 0.93 a | 0.20 ± 0.05 a |
| Ψ < -0.7 MPa | | | | |
| WW | -0.83 ± 0.03 a | 3.54 ± 1.22 b | 0.65 ± 0.34 b | 0.06 ± 0.03 b |
| TD | -0.74 ± 0.02 a | 3.06 ± 0.96 b | 0.43 ± 0.12 b | 0.03 ± 0.01 b |
| SD | -0.78 ± 0.02 a | 9.13 ± 2.10 a | 1.76 ± 0.56 a | 0.15 ± 0.05 a |
